# Some Recent Investigations and Their Bearing on a Theory of the Causation of Dental Caries

**Published:** 1914-09-15

**Authors:** J. Sim Wallace


### SOME RECENT INVESTIGATIONS AND THEIR BEARING ON A THEORY OF THE CAUSATION OF DENTAL CARIES.

BY J. SIM WALLACE, M.D., D.Sc., L.D.S.

Read before the Odonto-Chirurgical Society of Scotland.

My first duty and pleasure is to thank the Council of the Odontological Society of Scotland for the honor they have done me in inviting me to read a paper before their Society at this particular meeting. It is a pleasant idea to invite one who has left his native land for years to return, even though it be for a day or two. It may not be altogether satisfactory in the cause of science; there are, however, other things perhaps more important which possibly it is well to take into consideration. It has been said by our national bard that "The heart's aye the pairt aye that keept us richt or wrang." If, then, in the goodness of your hearts you have chosen to invite one who has but few claims to be, and certainly no wish to be regarded as a scientist, to read a paper before your learned Society, you need not necessarily have done wrong, for even without the experimental paraphernalia of the modern scientist, one who has simply a persistent desire to get at the truth may arrive far nearer it than the scientists who, as has recently been said, will not be present in heaven in such relatively large numbers as their less ambitious confreres.

The subject which I have chosen for my paper this afternoon is the relation more especially of mucus and saliva in the causation of dental caries.

### Mucus and Saliva.

For a considerable time it has been thought that mucus was in some way associated with the causation of caries. Thus Mr. Tomes says: "Young people are often brought to us in whom, coincidently with the extensive development of caries, we find an abundant flow of saliva, and a free secretion of mucus; but I think the latter is usually in excess, and is found clinging to the teeth, instead of becoming dissolved in the saliva." On the other hand, he mentions that cases "present themselves in which the teeth rapidly decay in mouths free from any increased vascularity, local or general—free from adherent mucus about the teeth, and also from any sign of that fluid being either excessive in quantity or vitiated in quality." And further, he notes that "the examination of the reaction of the saliva and of the buccal mucus in mouths where caries is rapidly progressive has hitherto disclosed discordant results."[1] Similarly Miller did not support the view that the viscosity of the saliva was important with regard to the causation of caries and instances cases of "immunes" in which the mucus was particularly viscid.

A few years ago[2] Dr. E. C. Kirk went into this subject a little more fully. He said that a "potent factor in its bearing upon the problem of susceptibility and immunity to caries is the reaction of the oral fluids. In the present state of our knowledge it is difficult to say with certainty what is the normal reaction of the mixed oral fluids, especially if by the term normal we imply an ideal standard of health with all the nutritive processes in perfect equilibrium. The mixed saliva is the combined product secreted by several pairs of special salivary glands which pour their secretion into the buccal cavity. In addition to this it contains the mucoid secretion of a multitude of mucous glands embedded at various points beneath the mucous membrane of the mouth. While a neutral or slightly alkaline reaction may be taken as normal to a healthy saliva, it is certain that where the saliva is mucoid in character, is viscid because it holds much mucin in solution, such a saliva is always alkaline in reaction, for the reason that mucin

1 Tomes, "A System of Dental Surgery," page 234, 4th edition.
2 The *Dental Cosmos*, 1910, page 733.

is precipitated by acids and by acid salts, consequently an acid saliva within certain limits is free of mucin, limpid and of thin consistence. My own observation of many cases as they are examined at our college clinic leads me to the conclusion that the most active expression of caries is to be found in mouths having an alkaline and highly mucoid saliva, and that such saliva is characteristic of the starch and sugar eater. We rarely find active caries in mouths having a thin limpid saliva of acid reaction. " "

These being Dr. Kirk's statements, it is evident that his views are not wholly in accord with those of Mr. Tomes. At one time I tested the reaction of the saliva between meals in cases in which caries was progressing rapidly and also in cases in which no caries had developed for at least a few years previously. I did not find that there was any constant relationship between the acidity or alkalinity of the saliva and the amount of caries present, and Miller's research evidently leads to the same conclusion. I came, in fact, to believe quite differently from Dr. Kirk, that is to say, I came to believe that the reaction of the resting saliva was not a potent factor in the causation of caries. Dr. Kirk also refers to mucin in relation to the bacterial plaque and says: "Assuming, then, a lactic acid bacterium temporarily fallen upon a protected area of tooth structure in a culture medium capable of supporting its vitality, and containing besides glucose a certain amount of mucin— all of which conditions are to be found in the saliva of the ordinary caries susceptible—fermentations of the glucose is at once set up, each molecule of the sugar being split up into two molecules of lactic acid, which is shed out into the surrounding medium, causing an immediate precipitation of mucin about the bacterial growth, which adheres to the tooth surface by virtue of the precipitated mucin acting as the binding material of the agglutinated mass." It is in this manner, Dr. Kirk believes, or at least believed, that localization of decay in protected areas originates in susceptible cases.

Possibly Dr. Kirk has given up the ideas just quoted and fallen in line with Tomes and Miller, as there does not seem to be any reference to this aspect of the subject in his recent communication to the *British Dental Journal* December 15th, 1913.

A point which the earlier investigators overlooked was the nature of the flow of the saliva during and for some minutes after the eating of food. As far as my own experiments with litmus go it would appear that during and immediately after the mastication of food, even though the food itself is alkaline in reaction—such as meat and chicken—the saliva is alkaline to litmus paper.

Until recently then, the role which mucus plays in the *prevention* of dental caries had not been considered, yet several of the facts upon which I based my explanation of the part which mucus plays had been established by physiologists. We were taught that the mucus lubricated the food bolus and the mucous membrane to facilitate the passage of the food to the stomach. Any function or effect which the mucus might have in regard to the teeth seems to have been overlooked. In 1900 I brought forward the idea that the mucus acted as a protective coating to the unrubbed surfaces of the teeth, that this mucous coating, although infested with micro-organisms, was normally of neutral reaction and that the micro-organisms facilitated its slow disintegration and removal. Evidence brought forward by Miller seemed to confirm experimentally the idea that such mucous coating, though infested by bacteria, was not likely under normal circumstances to decalcify the enamel. A few years later I further amplified the idea that this mucous coating on the teeth was normally of a protective nature in another way, by referring to the fact well known to physiologists that acids precipitated the mucin and that thus acid foods were prevented from doing injury to the enamel by the formation of a more or less impermeable barrier to the acid during its transient presence in the mouth while the afterflow of saliva, under such circumstances more especially, being of an alkaline character, the precipitated mucin was dissolved and washed away from the mouth.[3] It was obvious, however, that this mucous coating protected the necks or unrubbed part of the teeth and not the masticating surfaces; that the protection from caries or masticating surfaces of the teeth was brought about in a different way from the protection of the unrubbed parts to which the mucus clings. Further experimental evidence brought forward by Prof. Gies and his collaborators would still more tend to support this view of the functions of the mucus, for Prof. Gies shows that mucus which has been precipitated by acid is disorganized and consequently its removal facilitated (together no doubt with adherent food particles).

Here, as with regards to other points in the elucidation of the prevention of caries, a principle which has guided us is the nature of the food of primitive man and his ancestry. If the food is of a fibrous nature not only does the mucus cling to the fibrous shreds, but as these are swallowed, the mucus in the saliva, being of a more or less stringy nature, tends to rope down particles of food which might otherwise remain about the teeth. When, therefore, the foodstuffs are of what might be called a natural kind rather than of a viscid pastry or finely ground nature, the function of the mucus is purely beneficent, and it appears to be secreted in quality and quantity suitable to the requirements of the particular foods eaten. In America the role of the mucus has recently been subjected to extremely careful investigation and a view with regard to localization of dental caries by adhesive mucin masses has been brought forward differing from mine. My contention was, as you know, that the localization of caries depended upon the lodgeability of fermentable food in places which were liable from mechanical reasons more especially to procure their undue retention. I shall refer to the works of Dr. Gies and his collaborators, not only because of the thoroughness of their investigations,

3 Supplementary Essays on "The Cause and Prevention of Dental Caries," page 40.

but also because Dr. Gies has referred to the origin of the idea which led him to support a view similar to that of Dr. Kirk, previously referred to, but differing from mine. The origin of an idea often throws light upon its validity. Generally speaking, an idea explanatory of an erroneous assumption is liable itself to be erroneous. Dr. Gies suggested (1) that dental caries might be due to the action of micro-organisms upon carbohydrates on and between the teeth., *localized* in both cases by "adhesive mucin masses," or by other mechanical fixations. He also expressed the belief (2) that the disintegration of "adhesive mucin masses," or their prevention, might be an important feature of prophylactic treatment against dental caries; (3) that both disintegration and prevention might be accomplished satisfactorily with dilute acid; and (4) that "food acids" (the typical fruit acids and their acid salts) might be effectively used for such purposes. "These views were based on many years of experience in the work of precipitating mucus and mucoids, on the general physiological knowledge that salivary secretion is stimulated by fruit juices and in the opinion that acid fruit juices could do no damage to enamel during their *transient* appearance in the mouth." The origin of the views given in his own words are no doubt correct, but a perusal of the papers written by Dr. Gies and his collaborators indicates that they believed in what is still in America supposed to be a fact of the very greatest importance with regard to the prevalence of dental caries in some mouths and not in others, namely, the existence of susceptibility and immunity to dental caries. Thus, Doctors Gies and Lothrop, say: "That the *systemic condition* of the individual is an important factor in susceptibility to dental caries is a conviction that we cannot dismiss. Nevertheless, direct external attack upon teeth by micro-organisms appears to be the most important single factor in the carious processes. Mucinous plaques afford favorable conditions for such external attacks.[4] A year later Dr. Lothrop,

4 The Journal of the Allied Societies, December 1910, page 283.

with the counsel and guidance of Dr. Gies, which he acknowledges, as a true scientist does, said: "If it could be definitely established what forces are brought to bear in the production and maintenance of immunity, the problem of making decay a disease of rare occurrence would soon be solved."[5] It seems to me, therefore, and the nature of his researches also indicate it, that Drs. Gies and Lothrop were endeavoring to discover something in the mucous or oral secretion which migh account for susceptibility or immunity to caries. Dr. Gies believed he had found this something in adhesive mucin masses, and that the disintegration of these mucin masses or their prevention might be an important feature in the prophylactic treatment against dental caries. All that Dr. Gies brings forward however, to indicate that mucin masses might localize the action of micro-organisms on carbohydrates on or between the teeth, might, in my opinion, far better have been brought forward to show that the adhesive mucin masses, adhering as they do to the unrubbed surfaces of the teeth, would be most effectual in the *prevention* of the localization of carbohydrates in such situations, even when acid did not enter into the dietary. If the desire to find something which would localize carbohydrates had not existed, it certainly would not have occurred to anyone studying Dr. Gies and his collaborators' investigations that mucus would have such effect when it was *not* removed by "food acids." If the surfaces of the teeth were coated with mucin plaques *before* the eating of carbohydrates the mucous film would surely facilitate the removal of such carbohydrates rather than locate them. This, at least, was in part the view that I had of the function of the mucus adherent to the teeth admittedly with a fundamentally different idea from that which led Dr. Gies to his conclusions. That is to say, from other considerations, I had come to the conclusion that susceptibility and immunity were almost wholly mythical suppositions and that the normal reactions of the normal constituents of the oral

5The Journal of the Allied Societies, December, 1911, page 311.

secretions were all, if the diet was physiologically correct, beneficent in preventing dental caries. In the first report of their work Doctors Gies and Lothrop said: "Mucin occurs in saliva, and apparently also on dental surfaces, primarily as acid salts in concentrated colloidal solution. When viscid mucinous coatings are treated with basic material, such as carbonate of an alkali or an earthy element, the mucin mass becomes superficially more smeary and slippery by reason of the production of more soluble mucin salts *at the surface.* Complete mechanical removal of a mucin plaque from a tooth is facilitated by the addition of a *basic* material that renders the mucin superficially more vicous, but the slippery surface thus produced may make the application of considerable friction necessary for the detachment of the plaque. On the other hand, when a viscid, mucinous deposit is treated with *acid* material, the mucin mass is completely disintegrated by a curdling or agglutinative process, the particles are devoid of adhesiveness to smooth surfaces, stickiness disappears because of the precipitation of caseous mucin itself, and the entire disorganized mass may be readily flushed away."[6]

What could be more obvious, if the above facts be true, and without doubt they are, than that each and all of the properties of the mucus referred to are beneficent, inasmuch as they facilitate the removal of foodstuffs from the teeth? The superficial layers of the mucin mass are slippery at the surfaces before, during and immediately after the taking of food. How better could things be arranged, not for locating the food about the teeth, but for facilitating its removal from the mouth and teeth? Further, suppose carbohydrate did lodge upon the surface of the mucin plaques and produce acid, or if acid food itself did lodge upon the mucous coating on the teeth, and if the mucin mass is completely disintegrated and readily flushed away, does not this indicate that the function of the mucus is to clear away and not to localize such food? Further, I fail

6 Journal of the Allied Societies, December, 1910, page 283.

to see that Drs. Gies and Lothrop have brought forward any evidence to show that adhesive mucin masses are only present, or more prevalent in, the mouths of so-called susceptibles or that they have brought forward evidence to show that adhesive mucin masses are absent in so-called immunes. It would appear indeed that the reactions of the salivary secretions to the food are common to all, and if the food is physiologically correct we may dispense with the suppositions as to susceptibility and immunity which are still too generally supposed to exist. Now although I have indicated my belief that carbohydrates are not localized by adhesive mucin masses, but that they are localized by the stickiness or relatively lodgeability of certain foods (in spite of the normal self-cleansing processes) and by the form of the tooth and the nature of its surrounding parts, all the other points which Dr. Gies has brought forward are in no way antagonistic to my views, but indeed strongly support them. When the teeth are regular, as for example, in most children's temporary sets, adhesive or readily lodgeable food does not so frequently lodge about the surfaces of the teeth which are more or less continually coated with mucus, as they do in the crevices of the masticating surfaces. If masses of micro-organisms exist in such situations, that is to say, if lack of food requiring efficient mastication allows such masses to exist in such situations, finely ground pasty or viscous carbohydrates may well adhere to or rather become mixed up with such plaques and initiate a carious process. But the quality of the mucus is not what determines its lodgment, but the nature of the crevice, the method in which the food is impounded into the crevice and the adhesiveness of the foodstuff itself, together with other factors which I have alluded to elsewhere. It is well recognized that in mouths in which the mucus has become thick and stagnant for a long time, when the mucus is *never* rubbed away by the friction of mastication or removed by the acidity of the food, when moreover the mucous membrane is somewhat inflamed and the mucous secretion excessive, decay is very generally absent. It is rather where the mucus is frequently more or less effectually removed and where the mucus is brushed or rubbed off a part of the tooth which is liable to lodge carbohydrates, that we find caries; it is true also that where food of a specially sugary nature is habitually eaten the mucous membrane may be irritated and the secretion of mucus be profuse, and yet caries may be present, but this depends on the stickiness of the food itself, and the inefficacy of the self-cleansing processes to effect the complete removal of such food, notwithstanding an obvious attempt to do so.

The discordant inferences which have arisen from an analysis of the *resting* saliva would never have been made had it been recognized that it is the nature of the saliva and mucus secreted during the eating of a meal and for a short time after that is important. What experiments I have made on this would indicate that during and immediately after meals the saliva is invariably alkaline in reaction to litmus even although the foodstuff consumed is not acid. In a mouth which is for me most convenient for observation the resting saliva is invariably acid to litmus. So too is that flow of saliva which immediately precedes the taking of food into the mouth. Immediately meat, chicken and, of course, acid foods, are eaten, the saliva becomes alkaline. Indeed, the chewing of a tasteless tooth-pick promotes a flow of alkaline saliva. It is this secretion during the time of taking of food, and immediately after the taking of food, which is of by far the greatest importance in the removal of food which might be harmful to the teeth from the mouth. With regard to the faint acidity of the resting saliva or the faint alkalinity of the resting saliva, clinical observation does not appear to show any distinct advantage. No doubt the more alkaline the resting saliva the more easily or thoroughly would acid be neutralized. On the other hand, the more acid the saliva the more would the acid-forming bacteria be impeded in their growth. When it is recognized that the food may be of such a nature as to prevent a food mass lodging in a crevice or secluded spot from being permeated by, or washed away by saliva, we see that given the lodgment of a sticky and impermeable food mass the nature of the resting saliva will have little effect on the carious process, for the simple reason that the food mass excludes the saliva and may also be of such a nature as to hinder its activity (*e. g.*, the presence of much sugar).

It occurred to me that possibly the nature of the resting saliva might vary according to the nature of the diet habitually consumed. On many occasions after having questioned patients with regard to their diet, more especially having reference to the amount of acid consumed in fruit, vegetables and drinks, I tested the saliva with litmus, but found no constant relationship between the alkalinity or acidity of the resting saliva and the alkalinity or acidity of the food. It would appear to me that at present we would be treading very thin ice if we expected to be able to control the carious process by an attempt to control the acidity or alkalinity of the resting saliva. Indeed, in the present state of our knowledge it would be impossible to know what we really wanted; in other words, we hardly know whether a faint alkalinity or a faint acidity of the saliva is desirable for retarding the carious process. Moreover, the amount secreted during the resting state of the salivary glands, more especially during sleep, is so very small that even from this point of view the subject is hardly worth considering. We certainly do know beyond any possibility of doubt that if sticky carbohydrates are not washed out of the mouth before a patient goes to sleep, any saliva whatever will be an excellent culture medium for acid-forming micro-organisms.

When I enjoined caution in the use of alkaline mouth washes and later advocated that a mouth wash should be slightly acid, I did not intend that through exaggeration the effects would be claimed to be beneficial long after the effects of such mouth wash had passed completely.—*Dental Record.*

